# Balloon-Assisted Squid 12 Embolization of a Renal Angiomyolipoma With Imaging Findings Suggestive of Arteriovenous Communication: A Technical Report

**DOI:** 10.7759/cureus.114989

**Published:** 2026-08-22

**Authors:** Stavros Grigoriadis, Dimitrios Chalmoukis, Panagiota Maravitsa, George Magoufis, Konstantinos Palialexis, Stavros Spiliopoulos

**Affiliations:** 1 2nd Department of Radiology, Interventional Radiology Unit, University General Hospital Attikon, Athens, GRC; 2 Department of Nephrology, Ionion Clinic Ltd, Athens, GRC

**Keywords:** arteriovenous communicationl, balloon microcatheter, flow control, renal angiomyolipoma, squid 12, transarterial embolization

## Abstract

Renal angiomyolipomas (AMLs) are benign, hypervascular tumors that require transarterial embolization if ruptured or preemptively if at high risk of hemorrhage (>4 cm, intralesional aneurysms, childbearing age, symptomatic, etc.). The embolic materials of choice, in order to produce permanent occlusions of the distal microvasculature bed of the lesion, are microspheres and liquid embolic agents. Embolization may be technically challenging when imaging suggests arteriovenous (AV) communication because rapid flow can compromise controlled delivery of these agents. We report a 30-year-old woman with intermittent right-flank pain and a 5.0-cm right renal AML. Preprocedural computed tomography angiography demonstrated enlarged perilesional veins with early arterial-phase opacification, raising suspicion of intratumoral arteriovenous communication; however, a discrete arteriovenous fistula or high-flow shunt was not directly demonstrated on digital subtraction angiography. These findings prompted a balloon-assisted flow-control strategy to minimize the risk of reflux and non-target embolization. A Scepter™ Mini dual-lumen occlusion balloon microcatheter (Terumo Neuro, Aliso Viejo, CA, USA; balloon size 2.2×9 mm; distal/proximal outer diameter 1.6/2.8 Fr) was advanced into the dominant feeding artery over a 0.008-inch Traxcess™ Mini guidewire (Terumo Neuro, Aliso Viejo, CA, USA). After the 0.44-mL catheter dead space was primed with dimethyl sulfoxide, one 1.5-mL vial of Squid 12 (Balt, Montmorency, France) was injected slowly under temporary flow arrest. Completion angiography demonstrated complete tumor devascularization with preservation of uninvolved renal parenchymal perfusion, and immediate noncontrast computed tomography showed embolic material confined to the target vessels without visible venous migration. Moderate overnight postembolization pain was controlled with intravenous paracetamol, and the patient was discharged the following day. At three-month follow-up, magnetic resonance imaging demonstrated no residual enhancement and a reduction in calculated tumor volume from 49.5 to 11.6 cm³ (76.5%). This case demonstrates the technical feasibility of balloon-assisted Squid 12 embolization and highlights how preprocedural imaging can directly influence the endovascular strategy in selected complex renal embolization procedures.

## Introduction

Renal angiomyolipomas (AMLs) are benign mesenchymal neoplasms composed of variable proportions of adipose tissue, smooth muscle, and dysmorphic blood vessels. Most are sporadic and solitary, whereas a minority are associated with tuberous sclerosis complex and may be multiple and bilateral [1,2].

Macroscopic fat is the key imaging feature of classic AML. Chemical-shift magnetic resonance imaging (MRI) can further aid in the characterization through signal loss at fat-water interfaces, although fat-poor AMLs may remain difficult to distinguish from renal cell carcinoma [3].

Transarterial embolization is indicated for acute rupture or may be performed preemptively in selected high-risk tumors. However, treatment decisions should not rely on lesion diameter alone. Symptoms, lesion growth, intralesional aneurysm size, vascularity, patient characteristics, and access to follow-up should be considered. Selective arterial embolization is an established nephron-sparing option for ruptured or high-risk AMLs [4-6].

When imaging raises suspicion of AV communication, liquid or microparticle embolization may be complicated by reflux or unintended passage into the venous circulation [7]. Dual-lumen balloon microcatheters provide temporary flow arrest and can improve control during embolic agent delivery [8,9]. We describe balloon-assisted Squid 12 embolization of a symptomatic 5-cm renal AML with imaging findings suggestive of AV communication, emphasizing the use of a low-viscosity dimethyl sulfoxide (DMSO)-based liquid embolic agent delivered through a DMSO-compatible dual-lumen balloon microcatheter and highlighting how preprocedural computed tomography angiography (CTA) influenced device selection and procedural strategy.

## Technical report

Patient presentation and imaging

**Figure 1:**
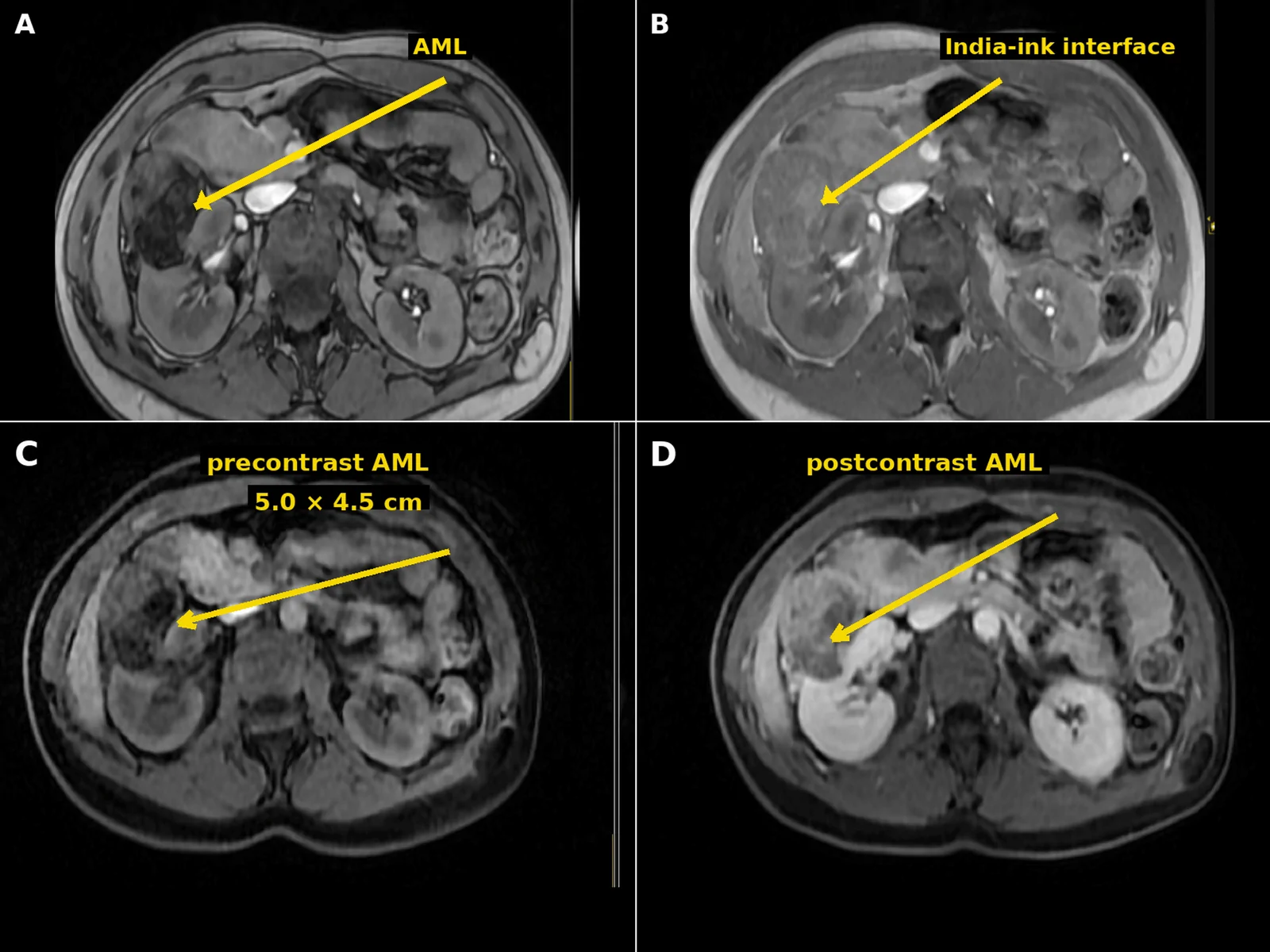
Preprocedural MRI The in-phase image identifies the right renal angiomyolipoma (A). The opposed-phase image demonstrates the characteristic India-ink artifact at the fat-water interface (B). On the displayed axial image, the lesion measures 5.0×4.5 cm. Precontrast (C) and postcontrast (D) fat-suppressed T1-weighted images show the intralesional macroscopic fat and enhancing soft-tissue component. Yellow arrows identify the lesion and the relevant imaging features. AML: angiomyolipoma; MRI: magnetic resonance imaging

A 30-year-old woman with no significant medical history presented with intermittent right-flank pain. MRI demonstrated a right renal mass containing macroscopic fat on in-phase imaging (Figure 1A), a characteristic India-ink artifact on opposed-phase imaging (Figure 1B), and the intralesional macroscopic fat and enhancing soft-tissue component on precontrast and postcontrast fat-suppressed T1-weighted images (Figures 1C, 1D), consistent with a typical AML. The lesion measured 4.5×5.0×4.2 cm, corresponding to an estimated ellipsoid volume of 49.5 cm³. Given the size and symptomatic presentation, as well as the childbearing age of the patient and the hypervascularity of the lesion, selective arterial embolization was selected as a nephron-sparing treatment.

Preprocedural planning

**Figure 2:**
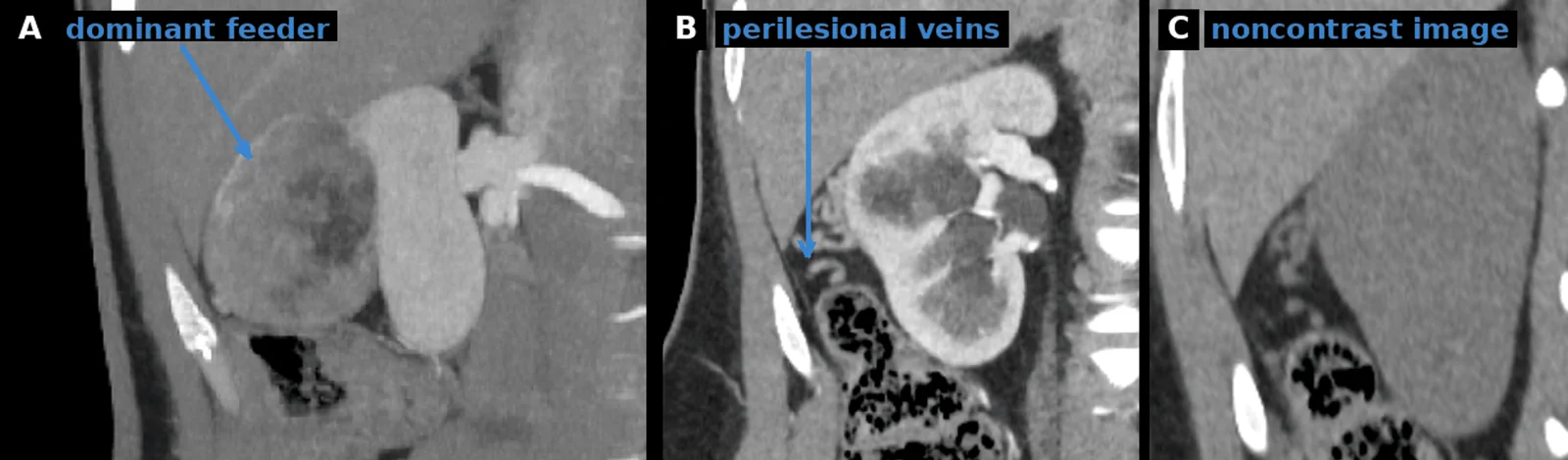
: Preprocedural CTA A multiplanar reformatted image demonstrates the dominant arterial feeder (A). Arterial-phase imaging demonstrates early opacification of enlarged perilesional veins, raising suspicion of intratumoral arteriovenous communication (B); however, a discrete arteriovenous fistula or high-flow shunt was not directly demonstrated. The corresponding noncontrast image is provided for comparison (C). Blue arrows identify the arterial feeder and perilesional veins. CTA: computed tomography angiography; AV: arteriovenous.

Preprocedural CTA was performed to delineate the arterial anatomy and plan super-selective access. CTA demonstrated a dominant tumor-feeding artery (Figure 2A) and enlarged perilesional veins in the perirenal fat caudal to the lesion, with early opacification during the arterial phase (Figure 2B), raising suspicion of intratumoral AV communication. However, no discrete arteriovenous fistula or high-flow shunt was directly demonstrated. The corresponding noncontrast CT image is shown for comparison (Figure 2C). These findings prompted adoption of a balloon-assisted flow-control strategy during liquid embolic delivery.

Embolization technique

**Figure 3:**
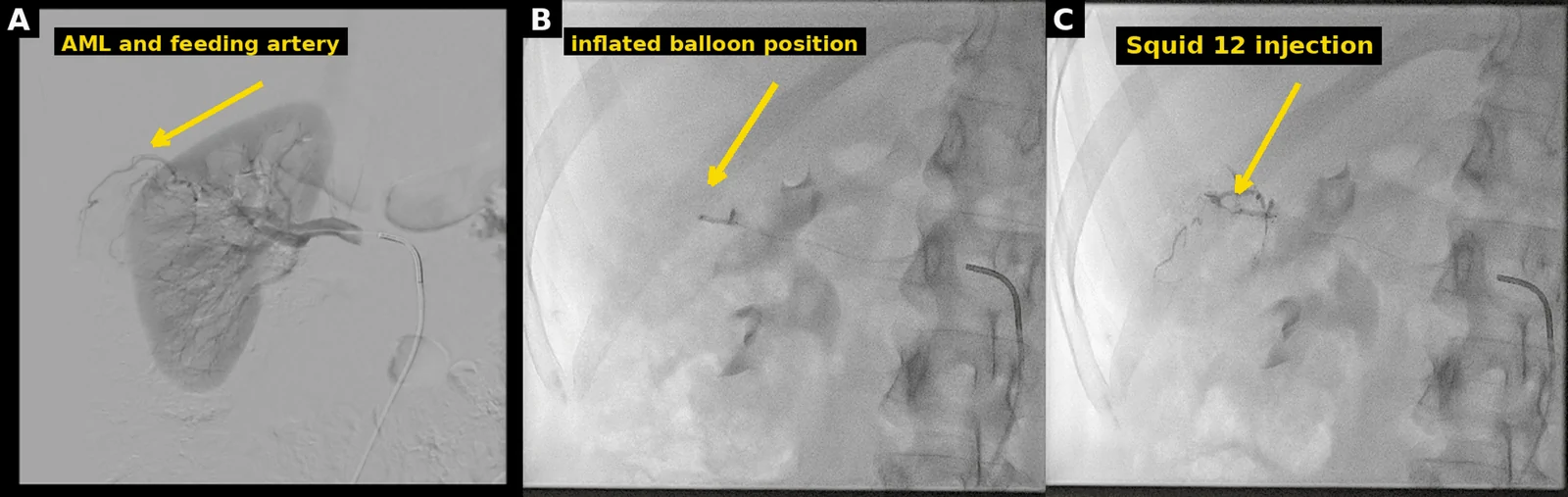
Balloon-assisted embolization Panel A demonstrates the angiomyolipoma and its dominant feeding artery before embolization. Panel B shows the balloon inflated at the super selective target position within the feeder. Panel C shows Squid 12 injection during balloon-assisted embolization. Yellow arrows identify the angiomyolipoma with its feeding artery, the inflated balloon position at the target site, and the Squid 12 injection, respectively.

Under local anesthesia and ultrasound guidance, right common femoral arterial access with a 5-Fr×11-cm arterial sheath was obtained, and a 5-Fr guiding catheter was positioned in the right renal artery. Digital subtraction angiography (DSA) confirmed the dominant tumor-feeding branch and the vascularity of the AML (Figure 3A). A dimethyl sulfoxide-compatible Scepter™ Mini dual-lumen occlusion balloon microcatheter (Terumo Neuro, Aliso Viejo, CA, USA; balloon size 2.2×9 mm; distal/proximal outer diameter 1.6/2.8 Fr) was advanced over a 0.008-inch Traxcess™ Mini guidewire (Terumo Neuro, Aliso Viejo, CA, USA) into the target feeder. Superselective angiography confirmed the treatment position and showed no definite early venous drainage under baseline flow conditions.

The balloon was gently inflated at the superselective target position to achieve temporary flow arrest and limit proximal reflux (Figure 3B). After the 0.44-mL catheter dead space was slowly primed with DMSO, 1.5 mL of Squid 12 (Balt, Montmorency, France) was slowly injected under continuous fluoroscopic monitoring while the balloon remained inflated (Figure 3C). Injection was discontinued after satisfactory intratumoral penetration and stasis were achieved. No significant reflux beyond the balloon or fluoroscopic evidence of venous migration was observed. Periprocedural prophylaxis consisted of cefuroxime 750 mg administered intravenously immediately before embolization and repeated the following day.

Outcome and follow-up

**Figure 4:**
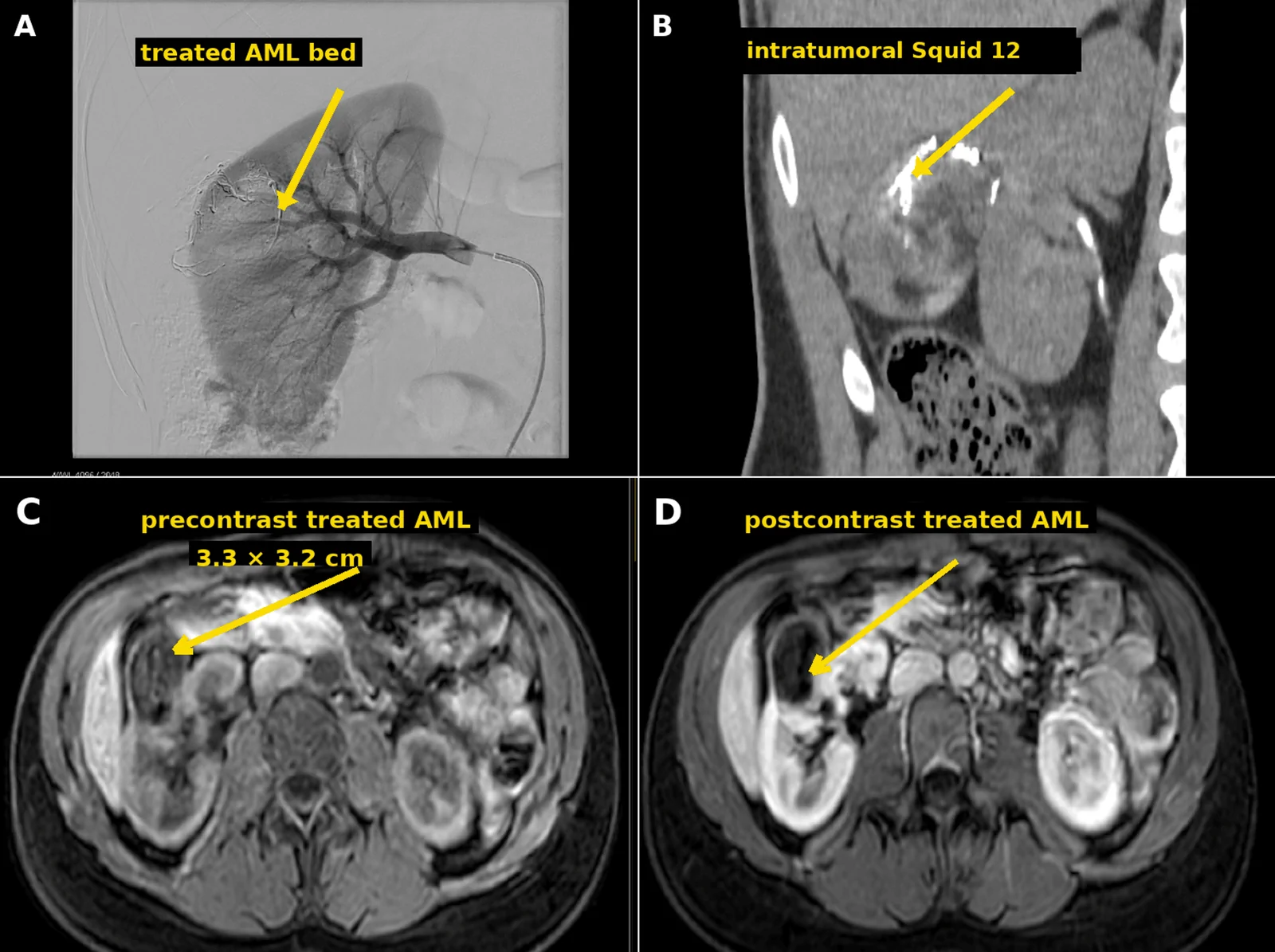
Procedural outcome and follow-up Completion DSA demonstrates the treated angiomyolipoma bed without residual tumor blush and with preservation of uninvolved renal parenchymal perfusion (A). Immediate noncontrast CT demonstrates radiopaque intratumoral Squid 12 without visible venous migration (B). At three-month follow-up, the displayed axial lesion dimensions are 3.3×3.2 cm on precontrast (C) and postcontrast (D) fat-suppressed T1-weighted MRI, with no residual enhancement. Yellow arrows identify the treated territory, embolic material, and residual treated lesion. DSA: digital subtraction angiography; CT: computed tomography; MRI: magnetic resonance imaging; AML: angiomyolipoma.

Completion DSA demonstrated complete angiographic devascularization of the AML with preservation of the remaining renal parenchymal perfusion (Figure 4A). Immediate noncontrast CT demonstrated radiopaque embolic material confined within the target tumor vessels, with no visible embolic material in the enlarged perilesional veins (Figure 4B). Serum creatinine was 1.2 mg/dL before embolization and remained unchanged at 1.2 mg/dL after the procedure. Three-month laboratory renal-function data were not available. The patient experienced moderate postembolization pain during the first postoperative night, which was controlled with intravenous paracetamol 1,000 mg; no additional analgesic medication was required. She was discharged asymptomatic the following day.

At three-month follow-up, MRI demonstrated a reduction in lesion dimensions to 3.3×3.2×2.1 cm on precontrast imaging (Figure 4C), corresponding to an estimated volume of 11.6 cm³ and a 76.5% reduction from baseline. Postcontrast imaging demonstrated no residual intralesional enhancement (Figure 4D). The patient reported complete resolution of the right-flank pain.

## Discussion

This report highlights the safety and efficacy of superselective embolization using a DMSO-compatible balloon-occlusion microcatheter and a low-viscosity DMSO-based liquid embolic agent to achieve controlled distal penetration while limiting reflux and unintended venous migration. It also emphasizes how preprocedural imaging may directly influence embolization strategy in selected renal AMLs. CTA did more than define the arterial anatomy: early opacification of enlarged perilesional veins raised suspicion of intratumoral AV communication, although a discrete AV fistula or high-flow shunt was not directly demonstrated angiographically. These findings prompted a balloon-assisted flow-control approach rather than conventional liquid embolization through a standard microcatheter.

Transarterial embolization is an established nephron-sparing treatment for symptomatic or high-risk AMLs. Contemporary systematic reviews confirm high technical and radiological success, although postembolization symptoms and the need for repeat intervention remain relevant considerations [5,6]. Reported embolic materials include particles, ethanol, coils, N-butyl cyanoacrylate, and ethylene-vinyl alcohol copolymers; material selection should be individualized according to vascular architecture, treatment objective, and operator experience.

Dual-lumen balloon microcatheters permit balloon occlusion and embolic delivery through separate lumens. In neurointerventional practice, this approach is used to modify local hemodynamics, facilitate controlled penetration of liquid embolic agents, and limit reflux [8,9]. Microballoon-assisted renal AML embolization has also been reported with ethanol-Lipiodol mixtures [10,11]. The present case extends this flow-control concept to Squid 12 in a lesion with suspected AV communication.

Temporary balloon occlusion created a more stable injection environment and allowed progressive intratumoral embolic deposition without significant reflux. Completion DSA showed preservation of uninvolved renal perfusion, immediate CT showed no visible venous migration, and three-month MRI demonstrated complete absence of residual intralesional enhancement with a 76.5% calculated reduction in tumor volume.

This case should not be interpreted as evidence that balloon assistance is routinely required for renal AML embolization or that this technique is superior to established alternatives. Most AMLs can be treated successfully using conventional superselective methods. Balloon-assisted delivery may be useful in selected lesions with suspected AV communication when the feeding artery can safely accommodate the device.

The principal limitations are the single-case design, short imaging follow-up, and indirect characterization of the suspected AV communication. Early venous opacification was demonstrated on CTA, but no discrete arteriovenous fistula or high-flow shunt was directly demonstrated angiographically or quantitatively established. Exact fluoroscopy, radiation-dose, and contrast-volume data were unavailable. Longer follow-up and larger series are required to assess reproducibility, safety, and durability. Nevertheless, this case illustrates how meticulous preprocedural vascular mapping may influence not only device selection but also the overall endovascular strategy in selected complex renal embolization procedures.

## Conclusions

Balloon-assisted Squid 12 embolization was technically feasible in this symptomatic renal AML with imaging findings suggestive of AV communication. Preprocedural CTA influenced treatment planning and supported the use of temporary flow arrest for controlled liquid embolic delivery, although a discrete AV fistula or high-flow shunt was not directly demonstrated angiographically. Complete tumor devascularization was achieved with preservation of renal parenchymal perfusion, and three-month MRI demonstrated marked volume reduction without residual intralesional enhancement. Although broader conclusions cannot be drawn from a single case, this approach may represent a useful technical option in carefully selected complex renal embolization procedures.
